# Risk prediction of atrial fibrillation and its complications in the community using hs troponin I

**DOI:** 10.1111/eci.13950

**Published:** 2023-01-17

**Authors:** Christin S. Börschel, Bastiaan Geelhoed, Teemu Niiranen, Stephan Camen, Maria Benedetta Donati, Aki S. Havulinna, Francesco Gianfagna, Tarja Palosaari, Pekka Jousilahti, Jukka Kontto, Erkki Vartiainen, Francisco M. Ojeda, Hester M. den Ruijter, Simona Costanzo, Giovanni de Gaetano, Augusto Di Castelnuovo, Allan Linneberg, Julie K. Vishram‐Nielsen, Maja‐Lisa Løchen, Wolfgang Koenig, Torben Jørgensen, Kari Kuulasmaa, Stefan Blankenberg, Licia Iacoviello, Tanja Zeller, Stefan Söderberg, Veikko Salomaa, Renate B. Schnabel

**Affiliations:** ^1^ Department of Cardiology University Heart and Vascular Centre Hamburg‐Eppendorf Hamburg Germany; ^2^ German Centre for Cardiovascular Research (DZHK) Partner Site Hamburg/Kiel/Lübeck Hamburg Germany; ^3^ Finnish Institute for Health and Welfare Helsinki Finland; ^4^ Deparment of Internal Medicine University of Turku Turku Finland; ^5^ Division of Medicine Turku University Hospital Turku Finland; ^6^ Department of Epidemiology and Prevention, IRCCS Neuromed Pozzilli Italy; ^7^ Institute for Molecular Medicine Finland (FIMM) Helsinki Finland; ^8^ Research Center in Epidemiology and Preventive Medicine (EPIMED), Department of Medicine and Surgery University of Insubria Varese Italy; ^9^ Mediterranea Cardiocentro Naples Italy; ^10^ Laboratory for Experimental Cardiology, University Medical Center Utrecht Utrecht University Utrecht The Netherlands; ^11^ Department of Clinical Medicine, Faculty of Health and Medical Sciences University of Copenhagen Copenhagen Denmark; ^12^ Centre for Clinical Research and Prevention Bispebjerg and Frederiksberg Hospital, The Capital Region Copenhagen Denmark; ^13^ Department of Cardiology, Rigshospitalet University of Copenhagen Copenhagen Denmark; ^14^ Department of Community Medicine UiT The Arctic University of Norway Tromsø Norway; ^15^ German Heart Centre Munich Technical University of Munich Munich Germany; ^16^ German Centre for Cardiovascular Research (DZHK) Partner Site Munich Heart Alliance Munich Munich Germany; ^17^ Institute of Epidemiology and Medical Biometry University of Ulm Ulm Germany; ^18^ Department of Public Health, Faculty of Health and Medical Sciences University of Copenhagen Copenhagen Denmark; ^19^ University Center of Cardiovascular Science University Heart and Vascular Center Hamburg‐Eppendorf Hamburg Germany; ^20^ Department of Public Health and Clinical Medicine Umeå University Umeå Sweden

**Keywords:** atrial fibrillation, biomarkers, epidemiology, high‐sensitivity troponin I, N‐terminal pro B‐type natriuretic peptide

## Abstract

**Aims:**

Atrial fibrillation (AF) is becoming increasingly common. Traditional cardiovascular risk factors (CVRF) do not explain all AF cases. Blood‐based biomarkers reflecting cardiac injury such as high‐sensitivity troponin I (hsTnI) may help close this gap.

**Methods:**

We investigated the predictive ability of hsTnI for incident AF in 45,298 participants (median age 51.4 years, 45.0% men) across European community cohorts in comparison to CVRF and established biomarkers (C‐reactive protein, N‐terminal pro B‐type natriuretic peptide).

**Results:**

During a median follow‐up of 7.7 years, 1734 (3.8%) participants developed AF. Those in the highest hsTnI quarter (≥4.2 ng/L) had a 3.91‐fold (95% confidence interval (CI) 3.30, 4.63; *p* < .01) risk for developing AF compared to the lowest quarter (<1.4 ng/L). In multivariable‐adjusted Cox proportional hazards models a statistically significant association was seen between hsTnI and AF (hazard ratio (HR) per 1 standard deviation (SD) increase in log10(hsTnI) 1.08; 95% CI 1.01, 1.16; *p* = .03). Inclusion of hsTnI did improve model discrimination (C‐index CVRF 0.811 vs. C‐index CVRF and hsTnI 0.813; *p* < .01). Higher hsTnI concentrations were associated with heart failure (HR per SD 1.37; 95% CI 1.12, 1.68; *p* < .01) and overall mortality (HR per SD 1.24; 95% CI 1.09, 1.41; *p* < .01).

**Conclusion:**

hsTnI as a biomarker of myocardial injury does not improve prediction of AF incidence beyond classical CVRF and NT‐proBNP. However, it is associated with the AF‐related disease heart failure and mortality likely reflecting underlying subclinical cardiovascular impairment.

## Graphical abstract

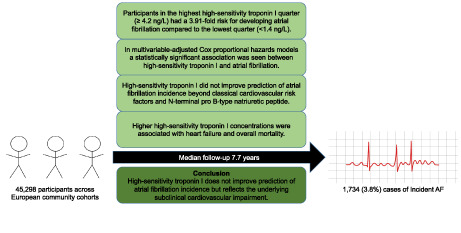

## 1 INTRODUCTION

Atrial fibrillation (AF) is increasingly common in the general population^1^ and associated with serious complications such as stroke and heart failure^2^, ^3^, ^4^, ^5^ as well as an overall higher mortality rate.^6^ Traditional cardiovascular risk factors (CVRF) do not entirely reflect an individual's risk for developing AF. Blood‐based biomarkers may help close this gap. To date, N‐terminal pro B‐type natriuretic peptide (NT‐proBNP), as an indicator of myocardial stress, and C‐reactive protein (CRP), which reflects inflammatory activity, have consistently been proposed as biomarkers for AF prediction.^7^ Highly sensitive troponin assays render the detection of very low troponin concentrations possible. Troponin is commonly used as a marker of myocardial injury in the setting of acute myocardial infarction. The novel highly sensitive troponin assays revealed an association between elevated troponin levels and a higher incidence of cardiovascular disease and cardiovascular mortality in the general population.^8^, ^9^, ^10^ Left ventricular hypertrophy, usually related to hypertensive blood pressure values, might also be reflected by higher troponin levels.^11^ Furthermore, troponin levels have been identified as predictors of incident AF in patients with cryptogenic ischemic strokes and in postoperative patients.^12^, ^13^ The association between elevated troponin concentrations and incident AF could also be confirmed in community‐based studies.^14^, ^15^, ^16^ Overall, however, the scientific evidence relating troponin I to incident AF is still limited and its incremental value as a risk marker over CVRF and biomarkers such as NT‐proBNP and CRP remains uncertain.

In this context, we investigated the predictive ability of high sensitivity troponin I (hsTnI) for the incidence of AF across different European populations from the Biomarkers for Cardiovascular Risk Assessment in Europe (BiomarCaRE) consortium in comparison to CVRF and the established biomarkers NT‐proBNP and CRP. We further examined the predictive ability of hsTnI for AF complications such as heart failure and cardiovascular events as well as overall mortality after newly diagnosed AF.

## 2 METHODS

### 2.1 Study population

Analyses were based on data of four European, population‐based, prospective cohort studies: FINRISK, Moli‐sani, MONICA Northern Sweden, and Scottish Heart Health Extended Cohort (SHHEC). All study cohorts are part of the Biomarker for Cardiovascular Risk Assessment in Europe (BiomarCaRE) consortium and were approved by the local ethics committees. Informed, written consent was obtained from all participants. A more detailed description of the studies has been published previously.^17^

In total, our study population comprised 45,298 individuals, of which 13.2% were from the FINRISK cohort, 44.9% from Moli‐sani, 18.6% from MONICA Northern Sweden, and 21.9% from SHHEC. Individuals with self‐reported and physician‐diagnosed history of AF as well as AF on the baseline ECG were defined as having prevalent AF and excluded from all analyses. Further, individuals with self‐reported and physician‐diagnosed history of heart failure, stroke, and cardiovascular disease including prior myocardial infarction were not included. Exclusion criteria and respective numbers are displayed in Table S1.

### 2.2 Data collection and biomarker measurements

At the time of inclusion in the studies, all participants completed a questionnaire, underwent physical examination, and provided a blood sample. The following risk factor information was collected: age, sex, body mass index (BMI), systolic and diastolic blood pressure, antihypertensive medication, smoking status, average alcohol consumption per day, diabetes status, serum‐lipid concentrations with different fasting duration across cohorts, as well as pre‐existing heart failure, coronary heart disease and stroke. Most variables were based on history. BMI and blood pressure were measured during the examination. During follow‐up, interim disease onset and outcomes were identified from national hospital discharge register partially including outpatient visits, national causes of death registers and the national drug reimbursement registers. The diagnosis of incident AF was based on study ECG tracings, questionnaire information, national hospital discharge registry data, ambulatory visits to specialized hospitals, and screening of death registry data. Regarding the Moli‐sani study, the assessment of events was made by record linkage with hospital discharge registers and national causes of death registers. The outcomes were AF, heart failure, stroke, cardiovascular events (including first fatal or non‐fatal myocardial infarction or stroke event) and total mortality. These data were harmonized for joint analysis in the MORGAM Project.^18^

Blood samples were stored at −80°C or in liquid nitrogen at −196°C (Moli‐sani) and freeze–thaw cycles were minimized. hsTnI concentrations were measured centrally in the BiomarCaRE core laboratory using a hsTnI immunoassay (Abbott Diagnostics, ARCHITECT i2000SR). NT‐proBNP levels were measured on the ELECSYS 2010 and the Cobas e411 using an electrochemiluminescence immuno‐assay (ECLIA, Roche Diagnostics) and high‐sensitive C‐reactive protein (hsCRP) was determined by latex enhanced immunoassay (Abbott, Architect c8000).

### 2.3 Statistical methods

Continuous variables were presented as median (25th, 75th percentile) and binary variables as absolute and relative frequencies. To study the associations of AF risk factors with time to AF, multivariable Cox regressions were performed. First, univariable Cox regression was performed to study the association between AF and of high sensitivity troponin I (hsTnI) quarter. Second, our primary multivariable model (Model I) included the following covariates not considered to be time‐varying and measured at baseline: sex, BMI, log10(serum triglycerides), alcohol consumption, systolic blood pressure, total cholesterol minus high density lipoprotein (HDL) cholesterol, current smoking, prevalent diabetes, and MORGAM geographic area (see Table S2) and with age as a time‐varying covariate (the analysis dataset was segmented into yearly time‐intervals and age was updated yearly using the start value it had at each interval). Time since baseline was used as the time scale in these Cox regressions. We selected the strongest biomarker from Cox regressions for further assessment of predictive ability in comparison with hsTnI. 12,036 of the 45,298 hsTnI values were below the assay range but were included in our analysis. Values of 0.0 ng/L were replaced by a small positive value (0.025 ng/L) in order to be able to perform log transformation. When hsTnI was included in a Cox regression, it was included as a continuous variable.

The primary model (Model I) was extended as follows: +log10(NT‐proBNP) (Model II), +log10(hsTnI) (Model III), and +log10(NT‐pro‐BNP) + log10(hsTnI) (Model IV). In Models I to IV, the linearity of the model was assessed by testing all possible second‐order interactions between covariates and adding them to the models I to IV if they reached significance in one or more models, taking into account multiple testing using Bonferroni correction. The proportional hazards assumptions were tested by looking at the correlation between transformed survival time and scaled Schoenfeld residuals as implemented by using the R function cox.zph()^19^ and interactions with time were added to the model if needed to make the proportional hazards assumption valid. If it was needed to add an interaction to one or more of the Models I to IV, it was automatically also added to the other models of Models I to IV. Continuous variables were always centred at their mean when included in an interaction. The ultimately obtained Models I to IV were tested with cox.zph() to be free of PH‐violations and also the Hosmer‐Lemeshow test showed no significant problems with the goodness‐of‐fits of these models. In supplemental analyses, we also fitted multivariable models without interactions. We performed multivariable models where only age, sex, and MORGAM geographic area remained as adjustments.

In secondary analyses, Cox regressions were performed for the hazard ratio (HR) of log10(hsTnI) for overall mortality, incident heart failure, incident cardiovascular disease, and incident stroke with the same adjustment as in our primary model (Model I).

Cumulative incidence curves where generated. The *p*‐value for the difference between cumulative incidence curves was calculated using the log rank test.

Harrel's C‐statistic, net reclassification improvement (NRI) and integrated discrimination improvement (IDI) were calculated for comparing the predictive performance of linear predictors calculated using the multivariable‐adjusted beta coefficients obtained in this study. The predictive abilities for incident AF were analysed using four different models: the beta coefficients of CVRF alone; the beta coefficients of CVRF and log10(hsTnI); the beta coefficients of CVRF and log10(NT‐proBNP); the beta coefficients of CVRF, log10(NT‐proBNP) and log10(hsTnI). Confidence intervals and *p*‐values for differences between C‐statistics were estimated using bootstrapping with 10,000 replications.^20^, ^21^ Each individual was repeated 10 times in the bootstrap population. Samples were drawn from this bootstrap population using simple random sampling.

A two‐sided *p*‐value of <.05 was considered statistically significant. Analyses were performed with R version 3.5.3.^22^

## 3 RESULTS

### 3.1 Baseline characteristics

The median age of the overall study cohort was 51.4 (lower and upper quartiles 43.1, 59.8) years, 45.0% (*N* = 20,375) were men. During a median follow‐up time of 7.7 (lower and upper quartiles 4.2, 17.3) years *N* = 1734 (3.8%) of the 45,298 participants developed AF. Median time to their first AF event was 11.8 (lower and upper quartiles 4.9, 17.4) years. In total 4108 participants died during the follow‐up time, which equals a mortality of 9.1%. Further baseline characteristics are shown in Table 1 as well as Tables S3 and S4.

**TABLE 1:** Baseline characteristics of the overall cohort.

	All (*N* = 45,298)
Age [years]	51.4 (43.1, 59.8)
Men No. (%)	20,375 (45.0)
Cardiovascular risk factors	
BMI [kg/m^2^]	26.4 (23.7, 29.6)
Total serum cholesterol [mmol/L]	5.7 (4.9, 6.5)
High‐density lipoprotein cholesterol [mmol/L]	96.0 (84.7–105.3)
Lipid‐lowering medication No. (%)	1726 (5.2%)
Systolic blood pressure [mm Hg]	133 (121,149)
Antihypertensive medication No. (%)	7854 (17.5)
Smoking no. (%)	11,058 (24.5)
Alcohol consumption per day [g]	4 (0, 15)
Diabetes no. (%)	1913 (4.2)
Biomarkers	
hsCRP [mg/L]	1.4 (0.6, 2.9)
NT‐proBNP [ng/L]	46.8 (25.0, 85.5)
hsTnI [ng/L]	2.4 (1.4, 4.2)
eGFR [mg/dl]	96.0 (84.7–105.3)

*Note*: Provided are median, 25th and 75th percentile for continuous variables. Number and percent are shown for categorical variables.

Abbreviations: BMI, body mass index; eGFR, estimated glomerular filtration rate; hsCRP, high‐sensitivity C‐reactive protein; hsTnI, high‐sensitivity Troponin I; NT‐proBNP, N‐terminal pro B‐type natriuretic peptide.

### 3.2 AF prediction models

**FIGURE 1:**
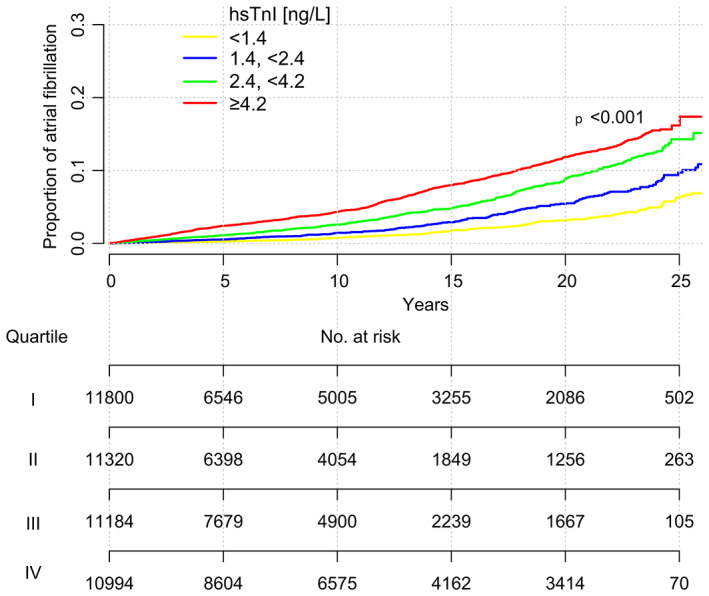
Cumulative incidence curves for atrial fibrillation (AF) by quartiles of high‐sensitivity measured troponin I (hsTnI). The respective numbers at risk are noted below.

The study population was divided into four equally sized groups depending on hsTnI values. In univariable Cox regression, those in the highest quarter of hsTnI values at baseline (≥4.2 ng/L) had a 3.91‐fold (95% confidence interval [CI] 3.30, 4.63; *p* < .01) higher hazard for developing AF compared to those in the lowest quarter (<1.4 ng/L). Cumulative incidence curves for AF by quarters of hsTnI and the respective numbers at risk are displayed in Figure 1.

**FIGURE 2:**
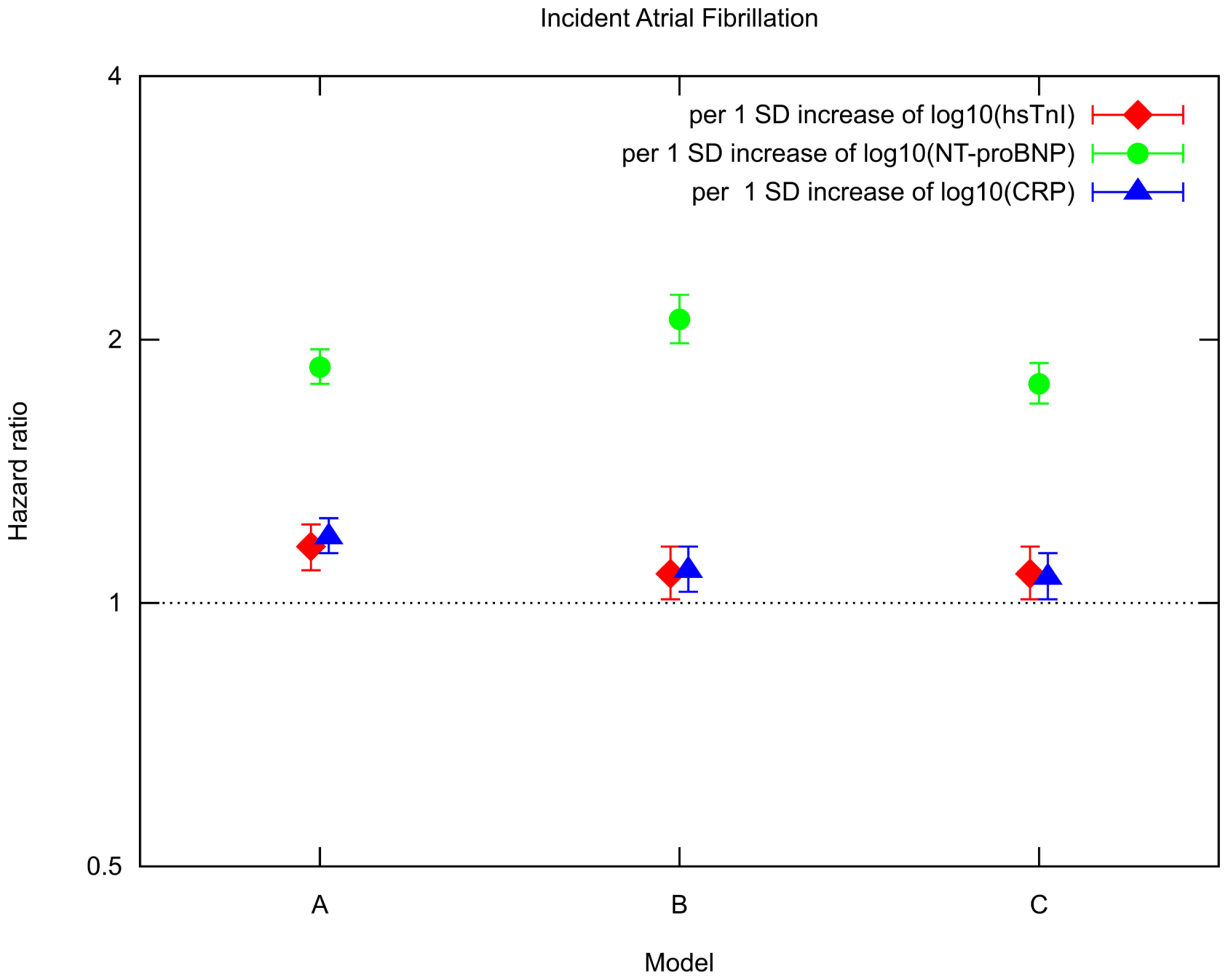
Predictive ability of log10(high‐sensitivity Troponin I [hsTnI]) and log10(N‐terminal pro B‐type natriuretic peptide [NT‐proBNP]) for incident atrial fibrillation (AF) in Cox regression model A: adjusted for age and sex and MORGAM geographic area; model B: multivariable‐adjusted for age, sex, BMI, log10(serum triglycerides), alcohol consumption, systolic blood pressure, total cholesterol minus HDL cholesterol, smoking status, prevalent diabetes, and MORGAM geographic area; model C: multivariable‐adjusted with heart failure, cardiovascular event and stroke as time‐dependent variables. Log10(HsTnI) was additionally adjusted for log10(NT‐proBNP) in models A, B and C. Provided are hazard ratios and 95% confidence intervals.

Cox proportional hazard models adjusted for age and sex revealed a significant association between log10(hsTnI) and AF (HR per 1 SD [=0.50] increase in log10(hsTnI) 1.16; 95% CI 1.09, 1.23; *p* < .01) and in multivariable‐adjusted Cox proportional hazard models also a statistically significant association was seen (HR per 1 SD [=0.50] increase in log10(hsTnI) 1.08; 95% CI 1.01, 1.16; *p* = .03). In multivariable‐adjusted Cox proportional hazard models log10(NT‐proBNP) (HR per 1 SD [=0.41] increase in log10(NT‐proBNP) 2.11; 95% CI 1.98, 2.25; *p* < .01) as well as log10(hsCRP) (HR per 1 SD [=0.48] increase in log10(hsCRP) 1.09; 95% CI 1.03, 1.16; *p* < .01) were statistically significantly related to incident AF. Hazard ratios for hsTnI, NT‐proBNP and hsCRP in different Cox proportional hazard models are presented in Figure 2 and Table S5.

For CVRF alone, the C‐index was 0.81 with bootstrapped standard deviation of 0.01. For the model comprising CVRF and log10(hsTnI) improvement of risk prediction compared to CVRF alone was found. Addition of log10(NT‐proBNP) as the strongest biomarker resulted in significantly improved model discrimination (C‐index 0.84, ΔC 0.026; 95% CI 0.019, 0.032; *p* < .001). The combination of CVRF and both biomarkers did not show higher predictive ability compared to the model with CVRF and log10(NT‐proBNP) alone. However, categorical NRI (with categories based on the quartiles of linear risk predictors) was highest (14.5%), when comparing the model with CVRF alone and the model with the combination of CVRF, log10(NT‐proBNP) and log10(hsTnI). IDI was highest (0.6218), when comparing the model with CVRF alone and the model with the combination of CVRF, log10(NT‐proBNP) and log10(hsTnI). C‐indices, NRIs and IDIs are shown in Table 2 and for each geographical area separately in Table S6.

**TABLE 2:** C‐indices, net reclassification improvement and integrated discrimination improvement for prediction of atrial fibrillation by hsTnI and NT‐proBNP in addition to cardiovascular risk factors.

	CVRF	CVRF, hsTnI	CVRF, NT‐proBNP	CVRF, NT‐proBNP, hsTnI
C‐indices	0.8111	0.8131	0.8367	0.8370
Improvement	—	0.0020	0.0256	0.0258
95% CI	—	0.0001, 0.0039	0.0189, 0.0323	0.0192, 0.0325
*p*‐Value	—	.044	<.001	<.001
NRI	—	0.047	0.143	0.145
IDI	—	0.1427	0.5773	0.6218

*Note*: Cardiovascular risk factors comprise age, sex, BMI, log10(serum triglycerides), alcohol consumption, systolic blood pressure, total cholesterol minus HDL cholesterol, smoking status, prevalent and diabetes.

Abbreviations: CI, confidence interval; CVRF, cardiovascular risk factors; hsTnI, high‐sensitivity Troponin I; IDI, integrated discrimination improvement; NRI, net reclassification improvement; NT‐proBNP, N‐terminal pro B‐type natriuretic peptide.

We further examined the predictive ability of log10(hsTnI) for overall mortality and the AF complications heart failure, cardiovascular events and stroke after newly diagnosed AF. Higher hsTnI concentrations were significantly associated with overall mortality and most prominent with heart failure (HR per 1 SD increase of log10(hsTnI) 1.37; 95% CI 1.12, 1.68; *p* < .01), as presented in Table 3.

**TABLE 3:** Predictive ability of log10(high‐sensitivity Troponin I (hsTnI)) for overall mortality and complications of atrial fibrillation (AF) after diagnosed AF in a multivariate Cox regression model.

	AF cases (*N*)	Events (*N*)	HR (95% CI) per 1 SD increase of log10(hsTnI)	*p*‐Value
Overall mortality	1332	411	1.24 (1.09, 1.41)	<.01
Heart failure	1094	165	1.37 (1.12, 1.68)	<.01
Stroke	1228	82	1.18 (0.88, 1.58)	.27
Cardiovascular event	1095	168	1.18 (0.95, 1.45)	.13

*Note*: Cardiovascular event includes: first fatal or non‐fatal myocardial infarction or stroke event. Adjustment comprised: age, sex, BMI, log10(serum triglycerides), alcohol consumption, systolic blood pressure, total cholesterol minus HDL cholesterol, smoking status, prevalent diabetes, and MORGAM geographic area.

Abbreviations: CI, confidence interval; HR, hazard ratio.

## 4 DISCUSSION

In our study we evaluated the applicability of hsTnI as a biomarker for AF prediction. Higher hsTnI concentrations were associated with a higher AF incidence. However, combining hsTnI and CVRF, there was no added predictive value over the model based on CVRF only. The addition of the more established biomarker NT‐proBNP improved AF risk prediction compared to CVRF alone. hsCRP showed borderline statistical significance in multivariable‐adjusted models. CVRF remained the strongest predictors of AF. Interestingly, hsTnI was associated with the AF complication heart failure as well as overall mortality indicating cardiac morbidity.

Our analysis of the predictive value of hsTnI in a large number of middle‐aged European participants provides new insights and adds to the still limited knowledge about the association between blood biomarkers and AF. We confirmed that the biomarker NT‐proBNP is a strong predictor of incident AF.^23^ The 76‐amino‐acid long amino‐terminal fragment of the precursor peptide of BNP is primarily released by the ventricular myocardium as a response to wall stress^24^ and thus related to heart failure. The close pathophysiological association between AF and heart failure consistently explains elevated levels of NT‐proBNP in AF. hsCRP as a marker of inflammatory activity has been shown to be related to incident AF, but its additional predictive value is known to be minor.^7^, ^25^

A pathophysiological link between hsTnI, as a marker of myocardial injury, and AF appears to be obvious. However, our analysis did not show a predictive value for AF beyond CVRF. An investigation of hsTnI and incident AF in the Framingham Heart Study revealed similar results to our study. Although hsTnI and incident AF were associated, they observed no improved discrimination or net reclassification beyond standard CVRF.^16^

Whereas hsTnI seems to be an excellent marker for ischemic cardiovascular events where it has been consistently associated with incident disease such as myocardial infarction or ischemic stroke,^8^, ^26^ pathophysiological mechanisms involved in the development of AF do not seem to be reflected strongly. The relation seems to be largely explained by CVRF.

In contrast, hsTnI may be a predictor of adverse outcomes in manifest AF as observed in our data. Our results showed that hsTnI is significantly associated with the AF complication heart failure as well as overall mortality after AF onset. In accordance, in a smaller community‐based study higher hsTnI was an independent predictor for hospitalization in the course of AF.^27^ In a Dutch study, hsTnI elevations in hospitalized patients with AF were shown to be associated with mortality and cardiac events.^28^ A retrospective analysis of about 1000 patients with newly diagnosed AF, revealed an association of higher hsTnI levels with a higher risk of all‐cause death as well as readmission rate for heart failure and revascularization.^29^ In addition, hsTnI levels may be useful as predictors of AF recurrence rate after ablation therapy of the pulmonary vein antrum.^30^ Thus, hsTnI may be a biomarker worth considering in manifest AF to predict outcomes and therapeutic success.

### 4.1 Strengths and limitations

The four included population‐based, prospective cohort studies are mainly composed of participants of European ancestry. Therefore, our findings may not be generalizable to other ethnicities. Likewise, the exclusion of individuals with previously diagnosed heart failure and myocardial infarction may limit generalizability. The outcomes were largely defined using national hospital‐based and drug reimbursement‐based databases. However, AF is often an outpatient diagnosis. Thus, we may have missed AF cases with outpatient diagnosis only. However, follow‐up during the last years also included out‐patient data and may have reduced this source of bias. A major strength of the study is the large number of individuals with harmonized baseline data across Europe in combination with a long follow‐up.

## 5 CONCLUSIONS

Our data suggest that myocyte stretch but not cellular injury may play a predominant role in AF. NT‐proBNP adds risk information for AF whereas the biomarker hsTnI did not further improve risk prediction for long‐term incidence of AF. A systematic approach combining CVRF and NT‐proBNP provided the most accurate risk prediction. hsTnI was associated with heart failure and overall mortality after AF onset and may be a biomarker for outcome risk assessment and therapeutic guidance.

## FUNDING INFORMATION

The BiomarCaRE Project was funded by the European Union Seventh Framework Programme (FP7/2007–2013) under grant agreement No. HEALTH‐F2‐2011‐278913. The MORGAM Project has received funding from EU projects MORGAM (Biomed, BMH4‐CT98‐3183), GenomEUtwin (FP5, QLG2‐CT‐2002‐01254), ENGAGE (FP7, HEALTH‐F4‐2007‐201413), CHANCES (FP7, HEALTH‐F3‐2010‐242244), BiomarCaRE (FP7, HEALTH‐F2‐2011‐278913), euCanSHare (Horizon 2020, No. 825903) and AFFECT‐EU (Horizon 2020, No. 847770); and Medical Research Council, London (G0601463, No. 80983: Biomarkers in the MORGAM Populations). This has supported central coordination, workshops and part of the activities of the MORGAM Data Centre, the MORGAM Laboratories and the MORGAM Participating Centers. The DAN‐MONICA cohorts at the Research Centre for Prevention and Health were established over a period of ten years and have been funded by numerous sources. The FINRISK surveys were mainly funded by budgetary funds of THL. Additional funding has been obtained from numerous non‐profit foundations. VS was supported by the Finnish Foundation for Cardiovascular Research. The Moli‐sani project was partially supported by research grants from Pfizer Foundation (Rome, Italy), the Italian Ministry of University and Research (MIUR, Rome, Italy)–Programma Triennale di Ricerca, Decreto n.1588 and Instrumentation Laboratory, Milan, Italy. The MONICA Northern Sweden study was supported by Norrbotten and Västerbotten County Councils, the Swedish Research Council (2011_2395), the Swedish Research Council for Health, Working Life and Welfare, the Swedish Heart and Lung Foundation (20140799, 20120631, 20100635) and the Joint Committee of the County Councils in Northern Sweden. RBS has received funding from the European Research Council (ERC) under the European Union's Horizon 2020 research and innovation programme under the grant agreement No 648131, from the European Union's Horizon 2020 research and innovation programme under the grant agreement No 847770 (AFFECT‐EU) and German Center for Cardiovascular Research (DZHK e.V.) (81Z1710103); German Ministry of Research and Education (BMBF 01ZX1408A) and ERACoSysMed3 (031 L0239). TN has received funding from the Academy of Finland (321351), the Emil Aaltonen Foundation, the Finnish Medical Foundation, the Paavo Nurmi Foundation, and the Finnish Foundation for Cardiovascular Research. KK reports grants from European Union, grants from Medical Research Council, UK, during the conduct of the study.

## CONFLICT OF INTEREST

RBS received consulting fees and speaker honoraria from BMS/Pfizer. SB reports grants and personal fees from Abbott Diagnostics, grants and personal fees from Bayer, grants and personal fees from SIEMENS, grants from Singulex, grants and personal fees from Thermo Fisher, personal fees from Astra Zeneca, personal fees from AMGEN, personal fees from Medtronic, personal fees from Pfizer, personal fees from Roche, personal fees from Novartis, personal fees from SIEMENS Diagnostics, outside the submitted work. VS reports personal fees from Novo Nordisk and Sanofi, grants from Bayer Ltd, outside the submitted work. SS reports participation in advisory boards (Actelion Ltd) and speakers' honoraria (Actelion Ltd and Bayer Ltd [unrelated to the present study]). WK reports advisory board fees from AstraZeneca, Novartis, Amgen, Pfizer, The Medicines Company, DalCor, Kowa, Corvidia, OMEICOS, Daiichi‐Sankyo, Novo Nordisk, New Amsterdam Pharma, TenSixteen Bio, Esperion, Genentech; lecture fees from Bristol‐Myers Squibb, Novartis, Amgen, Berlin‐Chemie, Sanofi and AstraZeneca; grants and non‐financial support from Abbott, Roche Diagnostics, Beckmann, and Singulex, all outside the submitted work.

## Supporting information


Figure S1.

Tables S1–S6.
