# Supplementary figures and images for: Risk prediction of atrial fibrillation and its complications in the community using hs troponin I
Source: Eur J Clin Invest. 2023 Jan 17;53(5):e13950. doi: 10.1111/eci.13950 (PMC11475262; doi:10.1111/eci.13950)

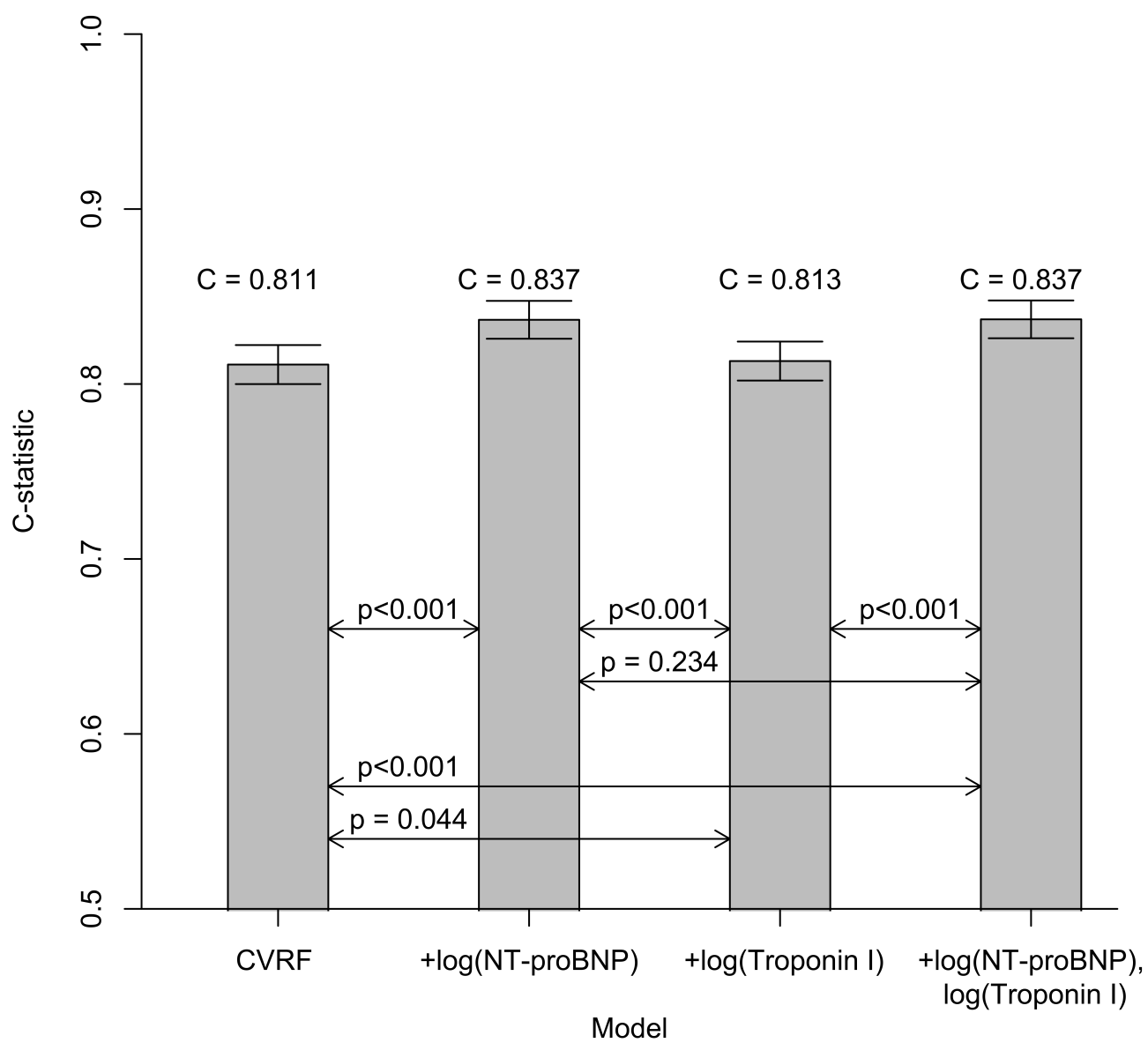

Supplement: Supplementary file 1 — Figure S1. [file ECI-53-e13950-s002.pdf]
