# Supplementary material for: Risk prediction of atrial fibrillation and its complications in the community using hs troponin I
Source: Eur J Clin Invest. 2023 Jan 17;53(5):e13950. doi: 10.1111/eci.13950 (PMC11475262; doi:10.1111/eci.13950)
Supplement: Supplementary file 2 — Tables S1–S6. [file ECI-53-e13950-s001.docx]

**Risk prediction of atrial fibrillation and its complications in the community using hs troponin I**

**Risk prediction of atrial fibrillation using hsTnI**

Christin S. Börschel^a,b^; Bastiaan Geelhoed^a,b^; Teemu Niiranen^c^; Stephan Camen^a,b^; Maria Benedetta Donati^d^; Aki S. Havulinna^c,e^; Francesco Gianfagna^f,g^; Tarja Palosaari^c^; Pekka Jousilahti^c^; Jukka Kontto^c^; Erkki Vartiainen^c^; Francisco M. Ojeda^a^; Hester M. den Ruijter^h^; Simona Costanzo^d^; Giovanni de Gaetano^d^; Augusto Di Castelnuovo^g^; Allan Linneberg^i,j^; Julie K. Vishram-Nielsen^j,k^; Maja-Lisa Løchen^l^; Wolfgang Koenig^m,n,o^; Torben Jørgensen^j,p,q^; Kari Kuulasmaa^c^; Stefan Blankenberg^a,b^; Licia Iacoviello^d,f^; Tanja Zeller^a,b^; Stefan Söderberg^r^; Veikko Salomaa^c^; Renate B. Schnabel^a,b^

^a^Department of Cardiology, University Heart and Vascular Centre Hamburg-Eppendorf, Hamburg, Germany; ^b^German Centre for Cardiovascular Research (DZHK), Partner Site Hamburg/Kiel/Lübeck, Hamburg, Germany; ^c^Finnish Institute for Health and Welfare, Helsinki, Finland; ^d^Department of Epidemiology and Prevention, IRCCS Neuromed, Pozzilli, Italy; ^e^Institute for Molecular Medicine Finland (FIMM), Helsinki, Finland; ^f^Research Center in Epidemiology and Preventive Medicine (EPIMED), Department of Medicine and Surgery, University of Insubria, Varese, Italy; ^g^Mediterranea Cardiocentro, Napoli, Italy; ^h^Laboratory for Experimental Cardiology, University Medical Center Utrecht, Utrecht University, Utrecht, The Netherlands; ^i^Department of Clinical Medicine, Faculty of Health and Medical Sciences, University of Copenhagen, Copenhagen, Denmark; ^j^Center for Clinical Research and Disease Prevention, Bispebjerg and Frederiksberg Hospital, The Capital Region, Copenhagen, Denmark; ^k^Department of Cardiology, Rigshospitalet, University of Copenhagen, Copenhagen, Denmark; ^l^Department of Community Medicine, UiT The Arctic University of Norway, Tromsø, Norway; ^m^German Heart Centre Munich, Technical University of Munich, Munich, Germany; ^n^German Centre for Cardiovascular Research (DZHK), Partner Site Munich Heart Alliance Munich, Munich, Germany; ^o^Institute of Epidemiology and Medical Biometry, University of Ulm, Ulm, Germany; ^p^Department of Clinical Experimental Research, Rigshospitalet, Glostrup, Denmark; ^q^Department of Public Health, Faculty of Health and Medical Sciences, University of Copenhagen, Copenhagen, Denmark; ^r^Department of Public Health and Clinical Medicine, Umeå University, Umeå, Sweden

All authors take responsibility for all aspects of the reliability and freedom from bias of the data presented and their discussed interpretation.

**Address for Correspondence**

Renate Schnabel, MD, MSc

University Heart and Vascular Centre Hamburg-Eppendorf

Department of Cardiology

Martinistr. 52

20246 Hamburg, Germany

Phone: 0049-1522-2816064

Email: r.schnabel@uke.de

**Supplemental Table 1.** Sequential exclusions.

| **Exclusion criteria** | **N before exclusion** | **N after exclusion** | **N excluded** |
| --- | --- | --- | --- |
| 1. Exclude individuals with baseline AF and/or missing baseline AF | 59245 | 58116 | 1129 |
| 1. Exclude individuals with baseline stroke and/or missing baseline stroke | 58116 | 57053 | 1036 |
| 1. Exclude individuals with baseline CVD and/or missing baseline CVD | 57053 | 55452 | 1601 |
| 1. Exclude individuals with baseline HF and/or missing baseline HF | 55452 | 55179 | 273 |
| 1. Exclude individuals with no FU | 55179 | 54752 | 427 |
| 1. Exclude individuals with missing baseline NT-proBNP | 54752 | 46258 | 8494 |
| 1. Exclude individuals with missing baseline hsTnI | 46258 | 45298 | 960 |

**Supplemental Table 2.** MORGAM geographical area used for adjustments.

| **Geographical area** | **N (%)** |
| --- | --- |
| Eastern and northern Finland | 3468 (7.7%) |
| Southern and western Finland | 2508 (5.5%) |
| Molise region in Italy | 20318 (44.9%) |
| Northern Sweden | 8404 (18.6%) |
| Edinburgh, Scotland | 711 (1.6%) |
| Glasgow, Scotland | 3385 (7.5%) |
| Rest of Scotland | 6504 (14.4%) |

Reference: https://www.thl.fi/publications/morgam/cohorts/

**Supplemental Table 3.** Baseline characteristics by cohort.

|  | **FINRISK**  **N=5,976** | **Moli-sani**  **N=20,318** | **Northern Sweden MONICA**  **N=8,404** | **SHHEC**  **N=10,600** |
| --- | --- | --- | --- | --- |
| Age [years] | 47.7 (36.9, 58.2) | 54.3 (45.8, 63.6) | 49.1 (37.8, 59.5) | 49.7 (43.4, 55.7) |
| Men No. (%) | 2743 (45.9%) | 9055 (44.6%) | 3678 (43.8%) | 4899 (46.2%) |
| *Cardiovascular risk factors* |  |  |  |  |
| BMI [kg/m^2^] | 25.9 (23.4, 28.9) | 27.4 (24.6, 30.6) | 26.1 (23.4, 29.4) | 25.3 (22.9, 28.0) |
| Total serum cholesterol [mmol/L] | 5.4 (4.7, 6.2) | 5.5 (4.8, 6.2) | 5.8 (5.0, 6.7) | 6.2 (5.4, 7.0) |
| High-density lipoprotein cholesterol [mmol/L] | 1.4 (1.2 - 1.6) | 1.4 (1.2 - 1.7) | 1.3 (1.1 - 1.6) | 1.5 (1.2 - 1.8) |
| Lipid-lowering medication No. (%) | 130 (3.1%) | 1331 (6.8%) | 255 (3.6%) | 10 (0.4%) |
| Systolic blood pressure [mm Hg] | 133 (121, 148) | 138 (125, 154) | 127 (115, 141) | 129 (117, 143) |
| Antihypertensive medication No. (%) | 684 (11.6%) | 5486 (27.2%) | 991 (11.9%) | 693 (6.5%) |
| Smoking No. (%) | 1316 (22.2%) | 4211 (20.7%) | 1549 (18.5%) | 3982 (37.6%) |
| Alcohol consumption per day [g] | 3.0 (0.0, 11.0) | 6.0 (0.0, 27.0) | 2.0 (0.0, 5.0) | 6.0 (0.0, 17.0) |
| Diabetes No. (%) | 295 (4.9%) | 1198 (5.9%) | 271 (3.2%) | 149 (1.4%) |
| *Biomarkers* | |  |  |  |
| hsCRP [mg/L] | 1.1 (0.5, 2.4) | 1.6 (0.8, 3.3) | 1.0 (0.5, 2.1) | 1.4 (0.6, 3.0) |
| NT-proBNP [ng/L] | 44.9 (24.1, 80.8) | 47.8 (25.6, 86.7) | 41.7 (21.7, 77.2) | 50.8 (27.3, 93.1) |
| hsTnI [ng/L] | 3.0 (2.0, 4.6) | 3.0 (1.4, 3.3) | 1.7 (0.9, 2.9) | 4.0 (1.9, 6.0) |
| eGFR [mg/dL] | 89.7 (77.8 - 101.4) | 94.6 (84.4 - 103.1) | 103.0 (92.2 - 113.1) | 96.7 (85.0 - 104.9) |

Provided are median, 25^th^ and 75^th^ percentile for continuous variables. Number and percent are shown for categorical variables.

Abbreviations: BMI, body mass index; eGFR, estimated glomerular filtration rate; hsCRP, high-sensitivity C-reactive protein; hsTnI, high-sensitivity Troponin I; NT-proBNP, N-terminal pro B-type natriuretic peptide; SHHEC, Scottish Heart Health Extended Cohort

**Supplemental Table 4.** Baseline characteristics by quartiles of high-sensitivity measured troponin I (hsTnI)

|  | **hsTnI Quartile 1**  **N=11800** | **hsTnI Quartile 2**  **N=11320** | **hsTnI Quartile 3**  **N=11184** | **hsTnI Quartile 4**  **N=10994** |
| --- | --- | --- | --- | --- |
| Age [years] | 44.9 (38.9 – 52.5) | 51.1 (42.9 - 59.0) | 55.1 (46.3 - 63.3) | 55.4 (47.6 - 63.6) |
| Men No. (%) | 2941 (24.9%) | 4823 (42.6%) | 6070 (54.3%) | 6541 (59.5%) |
| *Cardiovascular risk factors* |  |  |  |  |
| BMI [kg/m^2^] | 24.9 (22.5 – 28.0) | 26.6 (24.0 - 29.7) | 27.1 (24.5 - 30.4) | 27.0 (24.4 - 30.1) |
| Total serum cholesterol [mmol/L] | 5.3 (4.7 - 6.1) | 5.6 (4.9 - 6.4) | 5.8 (5.0 - 6.6) | 6.0 (5.2 - 6.9) |
| High-density lipoprotein cholesterol [mmol/L] | 1.5 (1.3 - 1.8) | 1.4 (1.2 - 1.7) | 1.4 (1.2 - 1.7) | 1.4 (1.2 - 1.7) |
| Lipid-lowering medication No. (%) | 265 (2.7%) | 487 (5.2%) | 541 (6.4%) | 433 (7.5%) |
| Systolic blood pressure [mm Hg] | 123.5 (114.0 - 135.5) | 132.5 (121.0 - 145.5) | 139.0 (126.0 - 153.5) | 433 (7.5%) |
| Antihypertensive medication No. (%) | 891 (7.6%) | 1856 (16.5%) | 2505 (22.6%) | 2602 (23.9%) |
| Smoking No. (%) | 2939 (24.9%) | 2638 (23.4%) | 2628 (23.6%) | 2853 (26.0%) |
| Alcohol consumption per day [g] | 2.0 (0.0 - 10.0) | 4.0 (0.0 - 14.0) | 5.0 (0.0 - 18.0) | 5.0 (0.0 - 18.0) |
| Diabetes No. (%) | 302 (2.6%) | 417 (3.7%) | 580 (5.2%) | 614 (5.6%) |
| *Biomarkers* | |  |  |  |
| hsCRP [mg/L] | 1.0 (0.5 - 2.3) | 1.3 (0.6 - 2.8) | 1.5 (0.7 - 3.1) | 1.6 (0.8 - 3.4) |
| NT-proBNP [ng/L] | 41.2 (23.2 - 68.9) | 42.3 (23.3 - 74.8) | 48.0 (24.8 - 89.6) | 61.5 (31.2 - 125.3) |
| hsTnI [ng/L] | 0.9 (0.5 - 1.2) | 1.9 (1.7 - 2.2) | 3.2 (2.8 - 3.6) | 6.2 (5.1 - 8.5) |
| eGFR [mg/dL] | 102.8 (93.8 - 110.6) | 96.9 (86.8 - 105.2) | 92.1 (81.3 - 101.6) | 90.0 (77.5 - 100.6) |

Provided are median, 25^th^ and 75^th^ percentile for continuous variables. Number and percent are shown for categorical variables.

Abbreviations: BMI, body mass index; eGFR, estimated glomerular filtration rate; hsCRP, high-sensitivity C-reactive protein; hsTnI, high-sensitivity Troponin I; NT-proBNP, N-terminal pro B-type natriuretic peptide

**Supplemental Table 5.** Cox regression models for incident atrial fibrillation.

**A** adjusted for age and sex, no interactions; **B** multivariable, no interactions; **C** multivariable, with interactions

|  | **A**  **HR (95% CI),**  **p-value** | **B**  **HR (95% CI),**  **p-value** | **C**  **HR (95% CI),**  **p-value** |
| --- | --- | --- | --- |
| Age (per 5-year increase) | 1.70 (1.65 - 1.75), <0.01 | 1.66 (1.61 - 1.72), <0.01 | 1.77 (1.68 - 1.86), <0.01 |
| Male sex | 1.60 (1.46 - 1.76), <0.01 | 1.58 (1.40 - 1.77), <0.01 | 2.19 (1.79 - 2.67), <0.01 |
| *Cardiovascular risk factors* |  |  |  |
| BMI (per 5 kg/m^2^ increase) | 1.35 (1.28 - 1.42), <0.01 | 1.36 (1.27 - 1.44), <0.01 | 1.34 (1.26 - 1.43), <0.01 |
| Systolic blood pressure (per 10 mm Hg increase) | 1.08 (1.06 - 1.11), <0.01 | 1.06 (1.03 - 1.09), <0.01 | 1.09 (1.05 - 1.13), <0.01 |
| Antihypertensive medication | 1.79 (1.59 - 2.01), <0.01 | 1.56 (1.35 - 1.79), <0.01 | 1.56 (1.35 - 1.79), <0.01 |
| Smoking | 1.15 (1.03 - 1.29), 0.01 | 1.28 (1.13 - 1.45), <0.01 | 1.28 (1.13 - 1.45), <0.01 |
| Alcohol consumption per week (per 10 g increase) | 1.06 (1.03 - 1.09), <0.01 | 1.05 (1.02 - 1.08), <0.01 | 1.04 (1.01 - 1.07), <0.01 |
| Diabetes | 1.36 (1.11 - 1.66), <0.01 | 1.29 (1.02 - 1.62), 0.03 | 1.31 (1.04 - 1.65), 0.02 |
| *Biomarkers* |  |  |  |
| Log10(hsCRP) (per 1 SD increase) | 1.19 (1.14 - 1.25), <0.01 | 1.09 (1.03 - 1.16), <0.01 | 1.09 (1.03 - 1.16), <0.01 |
| Log10(NT-proBNP) (per 1 SD increase) | 1.86 (1.78 - 1.95), <0.01 | 1.86 (1.76 - 1.96), <0.01 | 2.11 (1.98 - 2.25), <0.01 |
| Log10(hsTnI) (per 1 SD increase) | 1.16 (1.09 - 1.23), <0.01 | 1.10 (1.03 - 1.18), <0.01 | 1.08 (1.01 - 1.16), 0.03*  1.29 (1.21 - 1.38), <0.01** |
| Male sex * Age interaction (per 5-year increase) |  |  | 0.89 (0.84 - 0.94), <0.01 |
| Systolic blood pressure self-interaction (per 100 mm^2^ Hg^2^ increase increase) |  |  | 0.99 (0.98 - 1.00), <0.01 |

Abbreviations: BMI, body mass index; CI, confidence interval; HR, hazard ratio; hsCRP, high-sensitivity C-reactive protein; hsTnI, high-sensitivity Troponin I; NT-proBNP, N-terminal pro B-type natriuretic peptide

*with NT-proBNP adjustment; **without NT-proBNP adjustment

**Supplemental Table 6.** C-indices, net reclassification improvement and integrated discrimination improvement for prediction of atrial fibrillation by hsTnI and NT-proBNP in addition to cardiovascular risk factors for each geographical area separately.

|  | **CVRF** | **CVRF, hsTnI** | **CVRF, NT-proBNP** | **CVRF, NT-proBNP, hsTnI** |
| --- | --- | --- | --- | --- |
| **FINRISK** |  |  |  |  |
| C-indices | 0.8109 | 0.8064 | 0.8361 | 0.8352 |
| Improvement | - | -0.0045 | 0.0252 | 0.0243 |
| 95% CI | - | -0.0094, 0.0004 | 0.0133, 0.0370 | 0.0127, 0.0359 |
| p-value | - | 0.069 | <0.001 | <0.001 |
| NRI | - | -0.019 | 0.015 | 0.019 |
| IDI | - | 0.0859 | 0.6677 | 0.6945 |
| **Moli-Sani** |  |  |  |  |
| C-indices | 0.8369 | 0.8443 | 0.8680 | 0.8688 |
| Improvement | - | 0.0074 | 0.0311 | 0.0319 |
| 95% CI | - | 0.0042, 0.0106 | 0.0184, 0.0438 | 0.0192, 0.0446 |
| p-value | - | <0.001 | <0.001 | <0.001 |
| NRI | - | 0.025 | 0.049 | 0.049 |
| IDI | - | 0.2021 | 1.0645 | 1.1275 |
| **SHHEC** |  |  |  |  |
| C-indices | 0.7484 | 0.7537 | 0.7784 | 0.7797 |
| Improvement | - | 0.0053 | 0.0300 | 0.0313 |
| 95% CI | - | 0.0018, 0.0088 | 0.0184, 0.0417 | 0.0197, 0.0429 |
| p-value | - | 0.002 | <0.001 | <0.001 |
| NRI | - | 0.041 | 0.104 | 0.106 |
| IDI | - | 0.1240 | 0.4037 | 0.4423 |
| **SWE-NSW** |  |  |  |  |
| C-indices | 0.8247 | 0.8245 | 0.8411 | 0.8411 |
| Improvement | - | -0.0002 | 0.0164 | 0.0164 |
| 95% CI | - | -0.0036, 0.0031 | 0.0044, 0.0285 | 0.0046, 0.0281 |
| p-value | - | 0.884 | 0.007 | 0.006 |
| NRI | - | 0.000 | 0.025 | 0.025 |
| IDI | - | 0.1533 | 0.5407 | 0.5885 |

Abbreviations: CI, confidence interval; CVRF, cardiovascular risk factors; hsTnI, high-sensitivity Troponin I; IDI, integrated discrimination improvement; NRI, net reclassification improvement; NT-proBNP, N-terminal pro B-type natriuretic peptide.

Cardiovascular risk factors comprise age, sex, BMI, log10(serum triglycerides), alcohol consumption, systolic blood pressure, total cholesterol minus HDL cholesterol, smoking status, and prevalent diabetes.

**Supplemental Figure 1.** Bar graph of C-indices and 95% confidence intervals for atrial fibrillation (AF) prediction for classical cardiovascular risk factors (CVRF), and additionally the biomarkers N-terminal pro B-type natriuretic peptide (NT-proBNP) and high-sensitivity Troponin I (hsTnI) alone as well as in combination.
